# Accumulation and Clearance of Perfluorooctanoic Acid (PFOA) in Current and Former Residents of an Exposed Community

**DOI:** 10.1289/ehp.1002346

**Published:** 2010-09-22

**Authors:** Ryan Seals, Scott M. Bartell, Kyle Steenland

**Affiliations:** 1 Department of Environmental and Occupational Health, Emory University, Atlanta, Georgia, USA; 2 Program in Public Health and Department of Epidemiology, University of California, Irvine, California, USA

**Keywords:** C8, half-life, PFA, PFOA, serum levels, water contamination

## Abstract

**Background:**

Perfluorooctanoic acid (PFOA) is a perfluoroalkyl acid found in > 99% of Americans. Its health effects are unknown. Prior estimates of serum half-life range from 2.3 to 3.8 years.

**Objectives:**

We assessed the impact of years of residence and years since residing in the study area on serum PFOA concentration in a sample of current and former residents who were exposed to PFOA emissions from an industrial facility in six water districts in West Virginia and Ohio.

**Methods:**

Serum samples and questionnaires, including residential history, were collected in 2005–2006. We modeled log serum PFOA (nanograms per milliliter) for current residents as a function of years of residence in a water district, adjusted for a variety of factors. We modeled the half-life in former residents who lived in two water districts with high exposure levels using a two-segment log-linear spline.

**Results:**

We modeled serum PFOA concentration in 17,516 current residents as a function of years of residence (*R*^2^ = 0.68). Years of residence was significantly associated with PFOA concentration (1% increase in serum PFOA/year of residence), with significant heterogeneity by water district. Half-life was estimated in two water districts comprising a total of 1,573 individuals. For the participants included in our analyses, we found that years since residing in a water district was significantly associated with serum PFOA, which yielded half-lives of 2.9 and 8.5 years for water districts with higher and lower exposure levels, respectively.

**Conclusion:**

Years of residence in an exposed water district is positively associated with observed serum PFOA in 2005–2006. Differences in serum clearance rate between low- and high-exposure water districts suggest a possible concentration-dependent or time-dependent clearance process or inadequate adjustment for background exposures.

Perfluorooctanoic acid (PFOA, or C8) is a perfluoroalkyl acid used in the production of many fluoropolymers, including nonstick cookware, waterproofing, and flame retardants (Kennedy et al. 2004). Not naturally occurring, PFOA has been found in nature around the world, in multiple species, and in > 99% of serum samples obtained from the 2003–2004 National Health and Nutrition Examination Survey (Calafat et al. 2007; Houde et al. 2006). Despite recent regulatory and industrial efforts to phase out its production and use by 2015, PFOA accumulates and persists in the environment, and human exposure is not expected to cease for some time.

Studies in rodents suggest that PFOA may be associated with many disease outcomes, including increased hyperplasias and benign tumors of the testicles, liver, and pancreas, low birth weight, decreased immune response, and decreased cholesterol (Hines et al. 2009; Kennedy et al. 2004; Lau et al. 2007). However, the appropriateness of the animal models used in these studies has been called into question because of the wide range of clearance rates observed between and within species and because of species-specific differences in the role of the peroxisome proliferator-activated receptor alpha–mediated effects of PFOA (DeWitt et al. 2009; Lau et al. 2007; Rosen et al. 2009). Human studies of PFOA have been thus far largely limited to cross-sectional studies and retrospective analyses of occupational cohorts. To date no clear health effects of PFOA have been established, and studies on this topic are sparse.

Average concentrations of 3.9 ng/mL (equivalent to parts per billion) were found in a nationally representative sample of U.S. citizens in 2003–2004, with higher levels in males and whites (Calafat et al. 2007). Levels ranging from 100 to 5,000 ng/mL have been observed in occupational cohorts (Lau et al. 2007; Lundin et al. 2009; Olsen and Zobel 2007).

The current study population is derived from the C8 Health Project, which has been described previously (Frisbee et al. 2009). The C8 Health Project collected data on approximately 69,000 current and former residents of the mid-Ohio Valley who had been exposed to PFOA via contaminated drinking water. The average serum PFOA in this population was 82 ng/mL, with a median of 28 ng/mL (Steenland et al. 2009).

Establishing the rate of clearance of PFOA from the body is important to retrospectively determine lifetime exposure levels and to predict future serum concentrations. PFOA is known to persist in human serum long after exposure has ceased, and it is not metabolized in the body (Kennedy et al. 2004). Current estimates of serum half-life are derived from three primary sources. For 26 former employees of a manufacturing facility that produced PFOA, with an initial mean serum concentration of 799 ng/mL, Olsen et al. (2007) estimated an average half-life of 3.8 years [95% confidence interval (CI), 3.0–4.1], with individual half-lives ranging from 1.5 to 9.1 years based on a 5-year follow-up. In a more recent study of PFOA levels in 138 residents of Arnsberg, Germany, Brede et al. (2010) estimated a mean half-life of 3.26 years (range, 1.03–14.67 years) after charcoal filtration was installed in the community water system Finally, in an ongoing study of 200 community residents who were living near a PFOA facility and who were part of the C8 Health Project (a subset of the same population studied here) were followed for 1 year and, based on multiple blood samples and an initial mean serum concentration of 180 ng/mL, exhibited a half-life of 2.3 years (95% CI, 2.1–2.4) (Bartell et al. 2009). Half-life estimates based on only 1 year of follow-up must be considered with caution. Preliminary results indicate that a traditional exponential decay model is sufficient for describing the clearance of PFOA from the body, despite earlier indications that clearance may occur in a time-dependent fashion in animals (Tan et al. 2008).

Our goal in this study was to estimate the effect of duration of residence on PFOA levels among current residents and to estimate the effect of years since leaving among former residents. The latter goal also involved estimating half-life. Although using cross-sectional versus longitudinal data to estimate half-life is not optimal, it can provide useful information.

## Methods

### Data source

The C8 Health Project was conducted between August 2005 and August 2006. Health data were collected from current and former residents of the study area using an extensive questionnaire and blood test, including the serum concentration of PFOA (*n* = 69,030). A description of the study has been published previously (Frisbee et al. 2009). In addition to basic demographic information, the questionnaire included an extensive residential history beginning in 1980 and information on the source of drinking water at each address (public tap water, private well, bottled water). The questionnaire also queried individuals about such behaviors as smoking, alcohol consumption, and vegetarianism.

### Study participants

We identified individuals from the C8 Health Project who had consented to further follow-up and to release of identifiable data to us and who had provided residential history during the C8 Health Project (*n* = 48,880).

As noted, our goal was to study the effect of duration of residence in a water district and of years since leaving a water district on PFOA levels measured in 2005–2006. Ideally for our purposes, water within a district would have had a constant level of contamination over time, so that years of residence would reflect a constant exposure. In practice, however, PFOA emissions from the plant increased over time, peaking in the 1990s. In addition, different water districts are known to have had different levels of contamination, due largely to distance from the manufacturing plant (Steenland et al. 2009).

Because of likely high exposure levels at the chemical plant, we first excluded individuals who had a self-reported history of employment by DuPont (5%). We then excluded those who had a history of residence in more than one water district (25%), persons who ever reported using a private well as their primary source of drinking water (11%), or those who reported intermittent residence in the water districts in the study area (9%). These exclusions were included so that the subjects in our analyses had been continuously exposed to a single source of exposure within a single contaminated water district. Because the limit of detection for serum PFOA was 0.5 ng/mL, we also excluded individuals who were at or below this level (2%). Finally, we excluded individuals who reported overlapping residences in their residential history (3%). After all exclusions, 19,460 subjects remained for analysis.

### Current residents

Of the 19,460 subjects who were selected for our analyses, we identified 17,516 current residents who resided in one of the six water districts on the date of interview and testing. For the analysis of current residents, we focused on the effect of cumulative years lived in a water district.

### Former residents

We also studied a group of former residents to determine the effect of years since leaving a place of residence on seum-PFOA concentrations that were measured in 2005–2006 and to estimate the half-life of these concentrations. We limited our analysis of former residents to the two water districts of Little Hocking (Ohio) and Lubeck (West Virginia), because we hypothesized that these districts had higher levels of exposure. Among former residents, we excluded individuals with < 2 years residence in a water district (11%) and those with a serum PFOA concentration < 15 ng/mL (28%). These criteria were used to limit the analysis to individuals who had lived long enough in the water district to build up substantial levels of PFOA and who had sufficiently high baseline PFOA concentrations but that had not reached background levels of PFOA by the interview date. The final cohort of former residents consisted of 643 Little Hocking residents and 1,029 Lubeck residents.

This study was approved by institutional review boards at all C8 Science Panel institutions, and all applicable requirements for human research were met. All participants gave written informed consent to participate in the C8 Health project; consent procedures have been described previously (Frisbee et al. 2009).

### Statistical analysis

#### General models without exposure terms of interest

For all analyses, based on normalizing residuals for skewed data and in accordance with prior published results (Steenland et al. 2009), we modeled the natural logarithm of PFOA as measured in 2005–2006 as the outcome of a linear set of predictors. Variables considered as potential covariates were sex, age, race/ethnicity (white vs. nonwhite), body mass index (BMI), growing one’s own vegetables, vegetarianism, alcohol consumption, current and former smoking, regular exercise, and use of bottled water as primary source of drinking water. These variables were all measured in 2005–2006 and had been used in prior analyses of PFOA levels (Steenland et al. 2009). We used the categories described by Steenland et al. (2009) to define age, BMI, and date of interview.

For the analysis of current residents we used a backward selection process with a cutoff of 0.10 and without including duration of residence (our principal variable of interest) and created models individually for each of the six water districts to determine which covariates would be included in our final models. The backward selection process iteratively fit models, dropping the least-significant covariate at each step until all were significant at the cutoff level of 0.10. In the analyses with the six water districts combined, we added an indicator variable for water district to the model, which allowed us to determine the relative importance of residence in a particular water district as well as the effect of having resided in that district.

This process was repeated for the analysis of former residents, which was restricted to two water districts.

#### Analyses for duration of exposure

The goal of the first analysis was to estimate the relationship between duration of exposure to public water within a district and the measured serum PFOA level in 2005 or 2006. For this analysis we considered only individuals who had resided in the six water districts on the date of interview and testing (current residents). We analyzed cumulative years in the water district as both a continuous and categorical variable.


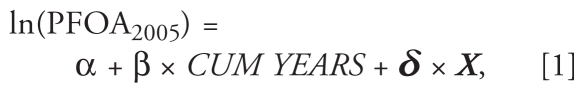


where *CUM YEARS* represents the number of years lived in the water district, and ***δ*** and ***X*** are parameter and covariate vectors.

#### Analyses by years since leaving a place of residence (half-life analysis)

A second analysis was performed to estimate the half-life in former residents only (restricted to two water districts), by analyzing the relationship between the number of years since living in the water district and the measured serum PFOA level in 2005 or 2006, using the following model:


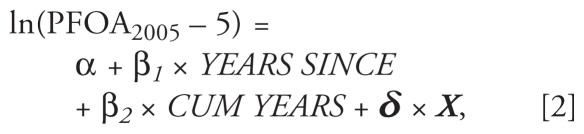


where *YEARS SINCE* represents the number of years that elapsed since residing in the water district, *CUM YEARS* is the number of years lived in the water district, and ***δ*** and ***X*** are parameter and covariate vectors. This analysis was restricted to the two water districts that had the highest levels, to avoid as much as possible the problem of background levels affecting our estimation of the elimination parameter (β). In this analysis, we subtracted background levels (5 ng/mL) from all subjects and required that all subjects had at least 15 ng/mL PFOA in 2005.

Although performed on a single cross-sectional measurement of serum PFOA, rather than the more traditional longitudinal analysis of repeated measurements, the analysis by years-since-leaving can provide an approximation of the clearance rate of PFOA. The number of years elapsed since living in the water district was analyzed as both a continuous and categorical variable.

The half-life of a serum concentration describes the number of years required for the concentration required to reach one-half of the baseline level. Elimination of a substance from the circulatory system is usually described by a logarithmic process, where the concentration of the substance at time *t* (C_t_) is related to the baseline concentration (C_0_) by the time-dependent term *e*^−λ^*^t^*, where λ is a positive decay constant, that is, *C_t_* *= C_0_e*^(−λ^*^t^*^)^. This is called a first-order elimination, in which the rate of elimination is constant and does not depend on initial concentration. To obtain the half-life (*t*_½_), we seek the time required such that *C_t_* is ½ of *C_0_*, or (*t*_½_), such that ½ × *C_0_* = *C_0_e*^(−λ^*^t^*^)^. Rearranging, we have


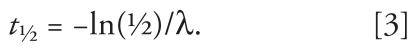


The slope of the line describing the relationship between *YEARS* and ln(PFOA) is β, which is equal to λ in Equation 3 above (also see below) and hence can be used to solve for the estimated number of years that would be required for the PFOA to fall by half. In equation 2 we have predicted PFOA = *e*^α^*e*^β × YEARS SINCE^*e****δ*^X^**, and because some change in *YEARS SINCE* will cut predicted PFOA in half, we have ½ = *e*^β × (YEARS SINCE1 – YEARS SINCE2)^, as the intercept and covariate terms cancel out. The change in YEARS SINCE is then the half-life, and taking the log of the last expression, we regain equation 3 and have


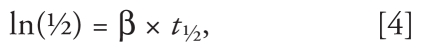


where β is equivalent to λ.

Graphing a scatter plot of log PFOA by YEARS SINCE, we found an apparent nonlinear relationship using a LOESS nonparametric curve. We then modeled the relationship using a two-segment linear spline (Steenland and Deddens 2004). The spline is included in the model through the addition of a time-dependent variable that is 0 prior to the knot and increases after the knot. Below is an example, with a knot at *YEARS SINCE = 4*:


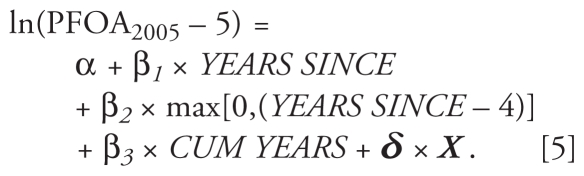


The expression max[0,(*YEARS SINCE –* 4)] evaluates to 0 when *YEARS SINCE* ≤ 4 and equals (*YEARS SINCE –* 4) when *YEARS SINCE* > 4. The slope of the regression line is therefore β_1_ prior to the knot and (β_1_ + β_2_) after the knot. We chose the knot based first on visual inspection of the relationship between years elapsed and ln(PFOA) to determine the likely region of interest, followed by an iterative procedure where we picked the knot with the best model likelihood. We used an F-test to test the increase in the goodness-of-fit in the spline model over the linear model. Outliers with absolute studentized residuals > 3 were discarded (0.5%).

Michalek et al. (1998) suggested that after truncation to account for near-background levels, half-life estimates can introduce bias. We performed a sensitivity analysis using various truncation values (serum PFOA concentrations below the truncation value were discarded) to assess the robustness of our half-life estimates, retaining the same models and knot locations as in the initial analysis with truncation at 15 ng/mL. In all analyses, 5 ng/mL was subtracted after truncation and before regression.

We performed all analyses using SAS (version 9.1; SAS Institute Inc., Cary, NC). Images were generated using PASW software (version 17.0; PASW, Chicago, IL).

## Results

Median levels of PFOA for current and former residents are shown in Table 1. In residents still residing in the six water districts at the time of the interview, differences in serum PFOA levels were apparent across water districts, sex, use of bottled water, growing own vegetables, smoking history, and date of testing, similar to results reported previously for the entire C8 Health Study cohort (Steenland et al. 2009). The subset of 1,672 former residents had higher PFOA levels than the current residents, because this subset was limited to residents of Little Hocking and Lubeck, the two highest-exposed water districts.

### Current residents

**Figure 1:**
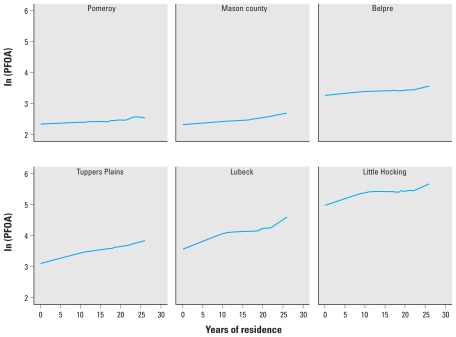
Plots of natural logarithm of PFOA (nanograms per milliliter) by cumulative years of residence in a water district, current residents, LOESS regression.

Figure 1 displays the relationship between cumulative years of residence in the six water districts and the natural logarithm of serum PFOA (nanograms per milliliter) in individuals reporting residence in one of the six water districts on the date of interview in 2005–2006 (current residents). The positive slope is significant at the *p* < 0.001 level for all six districts. The effect of cumulative years is reasonably linear with ln(PFOA).

The results of the full model after backward selection, with an indicator variable for water district, are shown in Table 2. The *R*-squared for the full model was 0.68. Water district residence explained the majority of the variance (partial *R*-squared), with residence in Little Hocking alone accounting for 39.4%. After residence, cumulative years of residence explained 1.5% of the variance. Previously observed associations were also replicated: higher levels in males, a U-shaped relationship with age, higher levels in current versus never smokers, and higher levels in those who grow their own vegetables (Steenland et al. 2009).

The average increase in PFOA levels for each year of residence in a water district was 1.2% (95% CI, 1.1–1.4%). However, because exposure levels are known to be different between water districts and because median serum PFOA levels differed so greatly by water district, we fit models for each water district separately to yield district-specific effects of cumulative residence (Table 3; F-test for six interaction terms significant at *p* < 0.001). As expected, districts with the highest exposure levels displayed the largest relationship between years of residential history and serum PFOA. In Pomeroy and Mason County, the districts with the lowest exposures as measured in current residents, the effect of years of residence was least.

### Former residents

**Figure 2:**
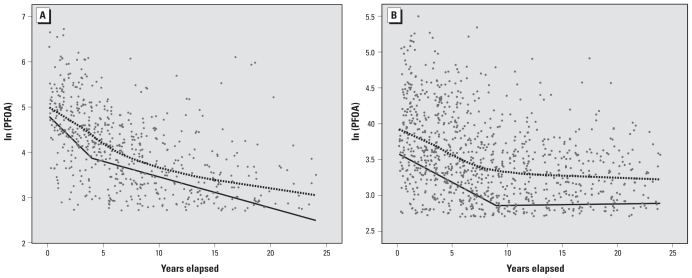
(*A*) Predicted decay of serum PFOA concentration based on half-lives estimated from former Little Hocking residents in discrete segments of < 4 and > 4 years since living in Little Hocking (solid line, adjusted for covariates) and LOESS regression (dashed line, unadjusted for covariates). (*B*) Predicted decay of serum PFOA concentration based on half-lives estimated from former Lubeck residents in discrete segments of < 9 and > 9 years since living in Lubeck (solid line, adjusted for covariates) and LOESS regression (dashed line, unadjusted for covariates).

Using a two-segment linear spline regression with the same variables as above, we obtained estimates for the effect of years elapsed since residence on ln(PFOA) for the two segments of the spline curve. Based on visual inspection of a LOESS curve and goodness-of-fit statistics comparing various possible knots, we chose 4 years as the knot for Little Hocking and 9 years as the knot for Lubeck. In Figure 2A and 2B, we show the plots and fitted lines for the two water districts. An F-test for reduction in model error after moving from one segment (standard linear regression) to two segments was significant at *p* < 0.001 for both water districts, and overall model fit (*R*^2^) increased by 4% in Little Hocking and 3% in Lubeck after inclusion of the spline. A three-segment linear spline was not a significantly better fit to the data in either water district.

The estimated half-lives and percent change in PFOA by year for the two line segments in each water district are shown in Table 4. Because the half-lives are calculated from the slope, they represent the half-life that would result if the instantaneous rate of clearance were to continue indefinitely. The shallower line after the knot likely reflects either the gradual slowing of PFOA clearance over time (Little Hocking) or the decline of low exposures to near background (15 ppb) in the case of Lubeck. For example, an individual from Little Hocking with an initial serum PFOA of 55 ng/mL would have a concentration of 21 ng/mL after 4 years (21% reduction per year) and a concentration of 15 ng/mL 4 years later (8% reduction per year). The resultant half-lives for Little Hocking were 2.9 and 10.1 years for the two spline segments, whereas for Lubeck they were 8.5 years in the initial spline segment and undefined for the second segment (the estimated parameter was 0.0021, or approximately 0, indicating no further decrease in PFOA levels over time). Our estimated half-lives were sensitive to the truncation cut point we used, below which subjects were excluded on the basis that they were near background levels. Table 5 displays various half-life estimates for the first spline segment in Little Hocking and Lubeck after various truncation values were applied. Little Hocking half-life estimates ranged from 2.5 to 3.0 years, whereas in Lubeck, estimates ranged from 5.9 to 10.3 years. At all truncation values, the half-life in Little Hocking was lower than in Lubeck, with larger discrepancies at higher truncation values. Because former Lubeck residents had lower serum PFOA concentrations, more individuals were discarded from Lubeck at all truncation values, with the discrepancy larger at higher values.

Our estimated half-lives were also sensitive to the amount we subtracted from our PFOA levels, a subtraction designed to eliminate background levels in estimating half-life. To test the robustness of our estimate to this subtraction, we performed a similar analysis that eliminated all individuals with values < 15 ng/mL in 2005 and subtracted 15 ng/mL, rather than 5 ng/mL, from their measured value. The results were similar to our original results: The estimated half-life for the first 4 years of clearance in former residents of Little Hocking was 3.0 years, whereas for Lubeck (for the first 9 years) it was 9.4 years.

PFOA clearance appears to be sex dependent in rats (with a much longer half-life in males) but not in monkeys (Lau et al. 2007). In our data for Little Hocking, males demonstrated a faster rate of clearance (*p* = 0.02) than did females, but only in the first 4 years. Annual reduction in serum PFOA was 27% in males versus 18% in females. The effect was nonsignificant after 4 years. However, prior longitudinal analyses of 200 residents in Little Hocking and Lubeck found no differences in half-life between the sexes (Bartell et al. 2009). We did not observe sex differences in former residents of Lubeck.

## Discussion

We found a significant positive association between years of residence in an exposed water district and serum PFOA, with an average of 1% increase per year of residence. Lower levels of serum PFOA in former versus current residents was demonstrated in this cohort previously (Steenland et al. 2009), but this analysis now demonstrates a significant trend within current residents (those still residing in exposed water districts in 2005–2006, based on their prior residential history). We also found a more substantial relationship between PFOA and years of residence in water districts closer to the industrial facility, as expected. After water district, years of residence accounted for the greatest variance in the fitted model. These findings provide preliminary justification for the possible use of residential history as a proxy for prior exposure in epidemiologic studies.

In former residents, the main finding from our analysis was that the use of a two-segment spline increased the model fit and better approximated the observed relationship than a simple linear model. In both water districts, an apparent nonlinear relationship resulted in a significantly lower clearance rate after the knot of either 4 or 9 years. If our assumptions are correct, this implies that a simple first-order elimination model may not hold and that the rate of elimination may be concentration dependent or time dependent. We feel that the results suggest both a concentration- and time-dependent relationship, because the time factor is the same for both Little Hocking and Lubeck (years since former residence), but exposure was lower in Lubeck. However, the apparent time-dependent relationship could also be due to the concentration decrease over time. It is interesting that the rate of decay (slope) of the second linear segment for Little Hocking is similar to the rate of decay for the first segment for Lubeck at similar concentration levels. In our cohort, former residents of Little Hocking had PFOA levels roughly twice as high as former residents of Lubeck. If serum clearance were concentration independent, the equation describing the relationship between PFOA and years living in the district and years elapsed since living in district would be the same in Little Hocking and Lubeck. Furthermore, within each water district, the decay in ln(PFOA) would be linear rather than exhibiting a lower slope at lower concentrations.

As in prior studies of this population, we observed decreasing serum concentrations across dates of testing (Steenland et al. 2009). This may be due to behavior modification as the putative health effects of PFOA became publicized, both in increased bottled water usage and decreased tap water consumption. We observed a slight increase in reported bottled water usage over the testing period, and Little Hocking was offering free bottled water to individuals. Additionally, it is plausible that those who tested earliest were those who lived closer to the industrial facility and in more highly exposed water districts. However, adjusting for date of testing did not significantly alter any of our parameters of interest.

In previous studies in humans, researchers have found no difference in clearance rates of PFOA between men and women, but animal studies have suggested that females rats may be more effective clearers of PFOA (Bartell et al. 2009; Brede et al. 2010; Lau et al. 2007). Harada et al. (2005) demonstrated in moderately exposed city dwellers that renal excretion rates in both males and females were negligibly small but that female clearance may be age dependent. In our cohort, we observed lower PFOA levels in females, an observation consistent with prior studies (Calafat et al. 2007; Steenland et al. 2009). However, we observed a significantly faster clearance rate in men in the first 4 years since moving away from Little Hocking. This calls into question the assumption that lower PFOA levels in females are due to faster rates of clearance, but we cannot rule out that the apparent sex effect may be due to concentration.

This study has three major limitations. The first is the cross-sectional nature of the analysis. Particularly in the estimation of half-life, this limited our ability to draw inferences from the analysis. Although cross-sectional half-life estimation has been used in an analogous setting for urinary bisphenol A after fasting (Stahlhut et al. 2009), traditional half-life studies follow individuals over time, allowing researchers to compare serum concentration at any point in time with the initial concentration. Cross-sectional analyses must rely on model-based estimation of the initial concentrations instead of directly observed values. Our regression model included years of residence in the contaminated water district, sex, age, growing own vegetables, smoking, and consuming bottled water. We relied on recall via questionnaire to develop a residential history. In addition to missing and incomplete data, such as gaps in residential history, which led to the exclusion of some subjects from the analysis, it is possible that individuals misreported their water district and years of residence.

The second major limitation is the implied assumption that exposure was uniform within a water district, both between individuals and over time, which we know to be false. Although we excluded individuals who were employed by DuPont or who reported private well use, to limit the heterogeneity of the population, individual exposure was undoubtedly varied based on geographical location, individual behavior, and other uncontrollable factors. In addition, we know that PFOA emissions from the plant were not constant over time and peaked in the late 1990s, but we were unable to account for this without quantitative estimates of annual water system concentrations. Further studies of this population will make use of advanced exposure models that account for both individual and temporal variations in exposure.

A third major limitation of our analysis is the potential bias introduced by the exclusion of participants with serum levels < 15 ng/mL. Truncation below a fixed concentration threshold is known to introduce bias in half-life estimates for longitudinal data (Michalek et al. 1998) and is likely to have a similar effect in cross-sectional analyses. Although restricting the analysis to individuals with PFOA serum concentrations < 15 ng/mL avoids one type of bias (overestimation of half-lives among participants whose PFOA serum concentrations are no longer in decline by the time of the serum sample), it is likely to introduce another type of bias resulting in overestimation of half-lives, because excluded participants are likely to have shorter half-lives on average than retained participants. Our sensitivity analysis using different truncation values resulted in a smaller range of values for the more highly exposed residents of Little Hocking, whereas the half-life in former Lubeck residents was more sensitive to the truncation value. Notably, Lubeck residents tended to have lower concentrations, so truncation at all values resulted in more individuals discarded from the Lubeck analysis, with a progressively larger difference at higher truncation values.

A minor limitation of this study was the inability to differentiate between variable exposure levels and accumulation due to constant exposure. However, because emission levels and predicted water concentrations were known to be variable over the study period, peaking in the late 1990s, we feel that some of the annual increase as shown by the significance of years of residence is likely due to increasing exposures rather than to a steady state (Paustenbach et al. 2007). Further work will be done with exposure estimates that vary by year and location of residence.

These results suggest that the half-life for PFOA lies between the previously reported estimates of 2.3 and 3.8 years for more highly exposed individuals, but that serum clearance of PFOA may be concentration dependent.

## Figures and Tables

**Table 1:** Median PFOA (nanograms per milliliter) in 2005–2006 (*n*) for current and former residents.

Variable	Current	Former^a^
Total	33.0 (18,068)	36.5 (1,672)

Water district		

Belpre	31.0 (1,999)	—
Little Hocking	241.0 (3,154)	60.6 (643)
Lubeck	69.4 (3,131)	31.0 (1,029)
Mason County	12.4 (5,052)	—
Pomeroy	11.8 (640)	—
Tuppers Plains	36.4 (4,092)	—

Sex		

Female	31.0 (9,330)	35.1 (872)
Male	34.9 (8,738)	37.3 (800)

Race/ethnicity		

White	32.9 (17,579)	36.5 (1,637)
Nonwhite	35.2 (489)	35.1 (35)

BMI (kg/m^2^)		

< 24	32.3 (6,328)	37.1 (575)
24–26	35.8 (3,454)	37.5 (354)
27–29	34.8 (3,099)	36.9 (315)
≥ 30	31.1 (5,187)	33.6 (428)

Regular exercise		

Yes	36.8 (5,794)	36.6 (645)
No	31.2 (12,274)	36.4 (1,027)

Drink bottled water		

Yes	55.5 (532)	77.7 (70)
No	32.9 (17,031)	36.2 (1,518)

Grow own vegetables		

Yes	38.3 (4,885)	39.4 (228)
No	31.5 (13,183)	35.9 (1,444)

Vegetarian		

Yes	37.1 (168)	38.0 (13)
No	33.0 (17,900)	36.5 (1,659)

Currently consume alcohol		

Yes	37.1 (6,108)	35.8 (845)
No	30.6 (11,216)	36.8 (784)

Smoker		

Current	28.1 (3,473)	35.5 (353)
Former	38.0 (3,968)	37.7 (364)
Never	33.1 (10,627)	36.6 (949)

Date of testing		

1 Aug–30 Sep 2005	59.7 (2,068)	39.2 (134)
1 Oct–30 Nov 2005	51.8 (2,377)	37.8 (218)
1 Dec 2005–31 Jan 2006	34.0 (5,389)	34.8 (514)
1 Feb–31 Mar 2006	28.7 (4,537)	35.3 (481)
1 Apr–31 May 2006	21.5 (2,431)	40.1 (147)
1 Jun–31 Aug 2006	17.0 (1,266)	38.0 (178)

a Former residents limited to individuals in Little Hocking and Lubeck with > 2 years residence and > 15 ng/mL PFOA.

**Table 2:** Multivariate linear regression results^a^ (*R*^2^ = 0.68), current residents (*n* = 17,516).^b^

Variable	Predicted change in PFOA (% from referent)	Log change in PFOA	95% CI	Variance (%) in ln(PFOA) (partial *R*^2^)
Cumulative years of residence	1	0.012	0.011 to 0.014	1.5
Sex, female	−12	−0.133	−0.153 to −0.112	0.9
Age (years)				
< 20	Referent	—	—	—
20–29	−23	−0.261	−0.302 to −0.220	0.9
30–39	−12	−0.126	−0.169 to −0.083	0.2
40–49	−1	−0.006	−0.045 to 0.033	0.0
50–59	4	0.042	0.004 to 0.080	0.0
60–69	18	0.167	0.126 to 0.208	0.4
> 70	27	0.236	0.192 to 0.279	0.6
Grow own vegetables	11	0.106	0.083 to 0.129	0.4
Smoking				
Never	Referent	—	—	—
Current	12	0.117	0.088 to 0.146	0.4
Former	1	0.012	−0.016 to 0.039	0.0
Drink bottled water	−26	−0.301	−0.361 to −0.241	0.5
Water district				
Tuppers Plains	Referent	—	—	—
Belpre	−12	−0.129	−0.166 to −0.091	0.3
Little Hocking	495	1.783	1.750 to 1.815	39.4
Lubeck	82	0.600	0.566 to 0.634	6.5
Mason County	−64	−1.018	−1.047 to −0.989	21.1
Pomeroy	−67	−1.102	−1.161 to −1.043	7.1

a Model also adjusted for date of visit.

b 552 individuals missing covariate data (bottled water = 505, smoking = 66).

**Table 3:** Effect of years of residence on serum PFOA by water district, with district-specific model fit.

Water district	*n*	Model *R*^2^	Variance (%) in ln(PFOA) (partial *R*^2^) explained by years of residence	Percent change in predicted PFOA by year of residence	95% CI
Tuppers Plains	3,986	0.18	3.2	1.7	1.4 to 2.0
Belpre	1,940	0.10	0.6	0.7	0.3 to 1.1
Little Hocking	3,054	0.10	0.8	1.2	0.7 to 1.7
Lubeck	3,044	0.23	3.6	1.9	1.6 to 2.3
Mason County	4,885	0.08	0.6	0.6	0.4 to 0.9
Pomeroy	607	0.12	0.5	0.5	−0.1 to 1.1

**Table 4:** Multivariate linear regression results, former residents of Little Hocking (*n* = 602)^a^ and Lubeck (*n* = 971).^b^

Variable	Estimated half-life (years)	Percent change in PFOA by year	95% CI
Little Hocking			
Years elapsed, < 4	2.9	−21.4	−26.1 to −16.5
Years elapsed, > 4	10.1	−7.6	−18.1 to 6.4
Years of residence	—	1.9	0.8 to 3.0
Lubeck			
Years elapsed, < 9	8.5	−7.8	−9.1 to −6.5
Years elapsed, > 9	n.a.^c^	0.2	−3.3 to 3.8
Years of residence	—	2.5	1.8 to 3.1

a Models also adjusted for sex, age, growing own vegetables, smoking, and consuming bottled water.

b Final analysis reflects missing data in smoking history and consumption of bottled water.

c Parameter (0.002) yields a positive half-life not significantly greater than zero.

**Table 5:** Sensitivity analysis for half-life after various truncation cut points of serum PFOA.

	Half-life [years (95% CI)]^a^	
Value (n/mL)	Little Hocking	Lubeck
20	3.0 (2.4–4.0)	10.3 (8.7–13.1)
15	2.9 (2.3–3.8)	8.5 (7.1–10.1)
10	2.5 (2.0–3.3)	6.6 (5.8–7.8)
5	2.7 (2.1–3.9)	5.9 (5.1–7.1)

a Models also adjusted for sex, age, growing own vegetables, smoking, and consuming bottled water.
